# *N*-*tert*-Butyl-3-mesitylpropanamide

**DOI:** 10.1107/S1600536811015856

**Published:** 2011-05-07

**Authors:** Abel M. Maharramov, Ali N. Khalilov, Atash V. Gurbanov, Iván Brito

**Affiliations:** a Department of Organic Chemistry, Baku State University, Baku, Azerbaijan; b Departamento de Química, Facultad de Ciencias Básicas, Universidad de Antofagasta, Casilla 170, Antofagasta - Chile

## Abstract

In the title compound, C_16_H_25_NO, the *N*-*tert*-butyl­propanamide fragment is essentially planar, with the exception of two C atoms of the *tert*-butyl group (r.m.s. deviation = 0.005 Å), forming a dihedral angle of 84.09 (10)° with the plane of the mesityl fragment (r.m.s. deviation = 0.002 Å). The crystal packing is stabilized by an inter­molecular N—H⋯O hydrogen bond, which links the mol­ecules into chains with graph-set notation *C*(4) running parallel to the *c* axis.

## Related literature

For graph-set notation, see: Bernstein *et al.* (1995 ▶).
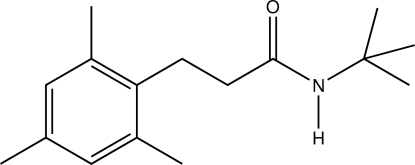


## Experimental

### 

#### Crystal data

C_16_H_25_NO
*M* *_r_* = 247.37
Monoclinic, 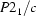

*a* = 12.8851 (11) Å
*b* = 13.3441 (11) Å
*c* = 9.4741 (8) Å
β = 106.540 (2)°
*V* = 1561.6 (2) Å^3^
*Z* = 4
Mo *K*α radiation
μ = 0.06 mm^−1^
*T* = 296 K
0.20 × 0.20 × 0.20 mm

#### Data collection

Bruker APEXII CCD diffractometer
Absorption correction: multi-scan (*SADABS*; Sheldrick, 2003 ▶) *T*_min_ = 0.987, *T*_max_ = 0.987
11870 measured reflections
3870 independent reflections
1738 reflections with *I* > 2σ(*I*)
*R*_int_ = 0.052

#### Refinement

*R*[*F* ^2^ > 2σ(*F* ^2^)] = 0.065
*wR*(*F* ^2^) = 0.173
*S* = 1.00
3870 reflections
169 parameters
H-atom parameters constrained
Δρ_max_ = 0.15 e Å^−3^
Δρ_min_ = −0.14 e Å^−3^

### 

Data collection: *APEX2* (Bruker, 2005 ▶); cell refinement: *SAINT* (Bruker, 2001 ▶); data reduction: *SAINT*; program(s) used to solve structure: *SHELXTL* (Sheldrick, 2008 ▶); program(s) used to refine structure: *SHELXTL*; molecular graphics: *SHELXTL* and *PLATON* (Spek, 2009) ▶; software used to prepare material for publication: *SHELXTL*.

## Supplementary Material

Crystal structure: contains datablocks I, global. DOI: 10.1107/S1600536811015856/om2425sup1.cif

Structure factors: contains datablocks I. DOI: 10.1107/S1600536811015856/om2425Isup2.hkl

Supplementary material file. DOI: 10.1107/S1600536811015856/om2425Isup3.cml

Additional supplementary materials:  crystallographic information; 3D view; checkCIF report

## Figures and Tables

**Table 1:** Hydrogen-bond geometry (Å, °)

*D*—H⋯*A*	*D*—H	H⋯*A*	*D*⋯*A*	*D*—H⋯*A*
N1—H1*N*⋯O1^i^	0.83	2.17	2.979 (2)	165

Symmetry code: (i) 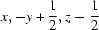.

**Fig. 1.**
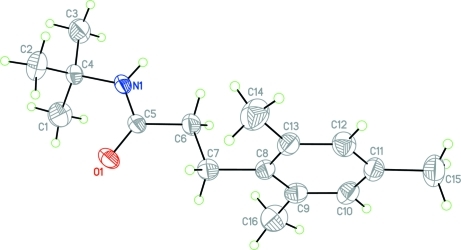
The molecular structure of the title compound, showing the atom labelling scheme. Displacement ellipsoids are drawn at the 30% probability level. H atoms are shown as circles of arbitrary radius.

**Fig. 2.**
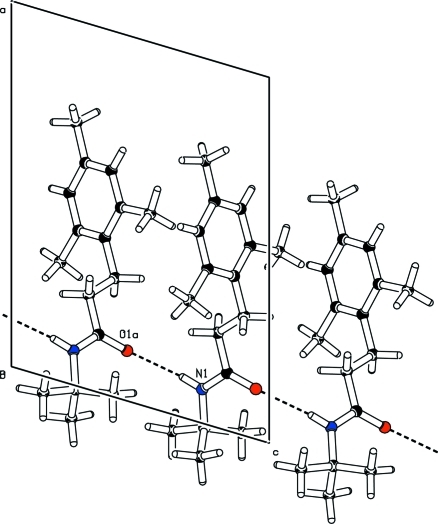
Part of the crystal structure showing the formation of a C(4) chain along [001]. Hydrogen bond shown as dashed lines. Symmetry code: (a) x, 1/2 - y, -1/2 + z.

## supplementary crystallographic information

### Comment

Fig. 1 shows the structure of title compound. Bond lengths and angles are unexceptional. The dihedral angle between the mesityl fragment and the C7/C6/C5/O1/N1/C4/C3 plane is 84.09 (10)°. Methyl groups of the benzene ring are into the same plane (r.m.s. deviation = 0.002 Å). In the crystal, molecules are linked by N— H···O interactions into chains with graph-set notation *C*(4) along [001], Figure 2, Table 1 (Bernstein *et al.*, 1995).

### Experimental

A mixture of 0.001 mol of 1-chloro-3-(2,4,6-trimethylphenyl)propan-2-one and 0.001 mol of *tert*-butylamine was stirred in water in presence of sodium hydroxide (0.003 mol) for 35–40 minutes. The crystals were recrystallized from ethanol solution (Yield 86%, melting point 143°C).^1^H NMRspectrum, DMSO-d_6_, δ, p.p.m..: 1.25 (s, 9H, 3CH_3_), 2.15 (s, 2H, CH_2_CO),2.25 (s, 9H, 3CH_3_), 2.75 (t, 2H, CH_2_Ar), 6.75 (s, 2H, 2CH_Ar_),7.45 (s, 1H, NHCO). ^13^C NMR spectrum, DMSO-d_6_, δ, p.p.m..: 19 (2CH_3_), 21 (CH_3_), 23(CH_2_CO), 25 [(CH_3_)_3_], 37 (CH_2_Ar),50 (C_i_), 129 (CH_Ar_), 136 (C_i_), 137 (C_i_),162 (CONH). IR spectrum, ν (cm^-1^)_:_ 3360,3170, 3005, 2928, 2878, 1645,1615, 1470, 1430, 715, 605.

### Refinement

All H-atoms were placed in calculated positions [C—H = 0.93 to 0.97 Å, *U*_iso_(H) =1.2 to 1.5 *U*_eq_(C) and N—H = 0.83 Å, *U*_iso_(H)=1.5 *U*_eq_(N)] and were included in the refinement in the riding model approximation. Due to weak diffracting ability of the crystal the ratio observed/unique reflections is low (45%).

### Crystal data

**Table:**

C_16_H_25_NO	*F*(000) = 544
*M**_r_* = 247.37	*D*_x_ = 1.052 Mg m^−^^3^
Monoclinic, *P*2_1_/*c*	Mo *K*α radiation, λ = 0.71073 Å
Hall symbol: -P 2ybc	Cell parameters from 1996 reflections
*a* = 12.8851 (11) Å	θ = 2.3–28.0°
*b* = 13.3441 (11) Å	µ = 0.06 mm^−^^1^
*c* = 9.4741 (8) Å	*T* = 296 K
β = 106.540 (2)°	Prism, colourless
*V* = 1561.6 (2) Å^3^	0.20 × 0.20 × 0.20 mm
*Z* = 4	

### Data collection

**Table:**

Bruker APEXII CCD diffractometer	3870 independent reflections
Radiation source: fine-focus sealed tube	1738 reflections with *I* > 2σ(*I*)
graphite	*R*_int_ = 0.052
φ and ω scans	θ_max_ = 28.3°, θ_min_ = 2.3°
Absorption correction: multi-scan (*SADABS*; Sheldrick, 2003)	*h* = −17→17
*T*_min_ = 0.987, *T*_max_ = 0.987	*k* = −15→17
11870 measured reflections	*l* = −12→12

### Refinement

**Table:**

Refinement on *F*^2^	Primary atom site location: structure-invariant direct methods
Least-squares matrix: full	Secondary atom site location: difference Fourier map
*R*[*F*^2^ > 2σ(*F*^2^)] = 0.065	Hydrogen site location: difference Fourier map
*wR*(*F*^2^) = 0.173	H-atom parameters constrained
*S* = 1.00	*w* = 1/[σ^2^(*F*_o_^2^) + (0.0732*P*)^2^ + 0.0483*P*] where *P* = (*F*_o_^2^ + 2*F*_c_^2^)/3
3870 reflections	(Δ/σ)_max_ = 0.001
169 parameters	Δρ_max_ = 0.15 e Å^−^^3^
0 restraints	Δρ_min_ = −0.14 e Å^−^^3^

### Special details

**Table:**

Geometry. All e.s.d.'s (except the e.s.d. in the dihedral angle between two l.s. planes) are estimated using the full covariance matrix. The cell e.s.d.'s are taken into account individually in the estimation of e.s.d.'s in distances, angles and torsion angles; correlations between e.s.d.'s in cell parameters are only used when they are defined by crystal symmetry. An approximate (isotropic) treatment of cell e.s.d.'s is used for estimating e.s.d.'s involving l.s. planes.
Refinement. Refinement of *F*^2^ against ALL reflections. The weighted *R*-factor *wR* and goodness of fit *S* are based on *F*^2^, conventional *R*-factors *R* are based on *F*, with *F* set to zero for negative *F*^2^. The threshold expression of *F*^2^ > σ(*F*^2^) is used only for calculating *R*-factors(gt) *etc*. and is not relevant to the choice of reflections for refinement. *R*-factors based on *F*^2^ are statistically about twice as large as those based on *F*, and *R*- factors based on ALL data will be even larger.

### Fractional atomic coordinates and isotropic or equivalent isotropic displacement parameters (Å^2^)

**Table:**

	*x*	*y*	*z*	*U*_iso_*/*U*_eq_	
O1	0.13712 (13)	0.29736 (13)	0.94818 (15)	0.0737 (5)	
N1	0.09333 (13)	0.20507 (13)	0.74037 (17)	0.0506 (5)	
H1N	0.1102	0.1935	0.6635	0.076*	
C1	0.0057 (2)	0.1074 (2)	0.8929 (3)	0.0898 (9)	
H1A	0.0617	0.0580	0.9039	0.135*	
H1B	−0.0606	0.0750	0.8940	0.135*	
H1C	0.0263	0.1545	0.9727	0.135*	
C2	−0.0933 (2)	0.2444 (2)	0.7318 (3)	0.0853 (8)	
H2A	−0.0668	0.2942	0.8065	0.128*	
H2B	−0.1597	0.2168	0.7417	0.128*	
H2C	−0.1062	0.2746	0.6364	0.128*	
C3	−0.0456 (2)	0.0878 (2)	0.6233 (3)	0.0851 (8)	
H3A	0.0075	0.0357	0.6355	0.128*	
H3B	−0.0529	0.1218	0.5316	0.128*	
H3C	−0.1140	0.0592	0.6231	0.128*	
C4	−0.01024 (17)	0.16181 (16)	0.7488 (2)	0.0521 (6)	
C5	0.15762 (17)	0.26650 (16)	0.8371 (2)	0.0510 (5)	
C6	0.25971 (18)	0.29603 (19)	0.8001 (2)	0.0676 (7)	
H6A	0.2405	0.3213	0.7000	0.081*	
H6B	0.3040	0.2368	0.8039	0.081*	
C7	0.32616 (18)	0.37485 (17)	0.9021 (2)	0.0607 (6)	
H7A	0.2826	0.4347	0.8977	0.073*	
H7B	0.3454	0.3500	1.0025	0.073*	
C8	0.42772 (17)	0.40167 (16)	0.8626 (2)	0.0505 (5)	
C9	0.5242 (2)	0.35047 (15)	0.9257 (2)	0.0570 (6)	
C10	0.61689 (19)	0.37656 (17)	0.8878 (2)	0.0611 (6)	
H10	0.6810	0.3426	0.9316	0.073*	
C11	0.61718 (18)	0.45079 (17)	0.7879 (3)	0.0587 (6)	
C12	0.52139 (19)	0.49934 (17)	0.7257 (2)	0.0630 (6)	
H12	0.5199	0.5495	0.6570	0.076*	
C13	0.42684 (17)	0.47697 (17)	0.7607 (2)	0.0572 (6)	
C14	0.3253 (2)	0.5357 (2)	0.6890 (3)	0.0936 (9)	
H14A	0.3410	0.5858	0.6252	0.140*	
H14B	0.2998	0.5676	0.7636	0.140*	
H14C	0.2706	0.4912	0.6326	0.140*	
C15	0.7201 (2)	0.4793 (2)	0.7516 (3)	0.0929 (9)	
H15A	0.7026	0.5186	0.6631	0.139*	
H15B	0.7576	0.4197	0.7374	0.139*	
H15C	0.7655	0.5177	0.8311	0.139*	
C16	0.5308 (2)	0.26788 (19)	1.0379 (3)	0.0885 (9)	
H16A	0.6026	0.2403	1.0664	0.133*	
H16B	0.4797	0.2162	0.9954	0.133*	
H16C	0.5144	0.2949	1.1229	0.133*	

### Atomic displacement parameters (Å^2^)

**Table:**

	*U*^11^	*U*^22^	*U*^33^	*U*^12^	*U*^13^	*U*^23^
O1	0.0738 (12)	0.1073 (13)	0.0501 (8)	−0.0259 (10)	0.0340 (8)	−0.0230 (9)
N1	0.0494 (11)	0.0637 (11)	0.0448 (9)	−0.0096 (9)	0.0234 (8)	−0.0049 (9)
C1	0.088 (2)	0.106 (2)	0.0798 (17)	−0.0271 (17)	0.0309 (15)	0.0263 (16)
C2	0.0554 (16)	0.092 (2)	0.112 (2)	0.0023 (14)	0.0289 (15)	−0.0023 (16)
C3	0.0772 (18)	0.098 (2)	0.0874 (18)	−0.0387 (16)	0.0342 (15)	−0.0283 (16)
C4	0.0465 (13)	0.0615 (13)	0.0528 (12)	−0.0089 (11)	0.0211 (10)	0.0006 (11)
C5	0.0506 (13)	0.0637 (14)	0.0426 (11)	−0.0100 (11)	0.0193 (10)	−0.0045 (11)
C6	0.0587 (15)	0.0892 (17)	0.0620 (13)	−0.0250 (13)	0.0287 (12)	−0.0247 (13)
C7	0.0588 (15)	0.0677 (14)	0.0581 (13)	−0.0112 (12)	0.0204 (11)	−0.0143 (12)
C8	0.0481 (13)	0.0538 (12)	0.0503 (11)	−0.0108 (11)	0.0151 (10)	−0.0124 (11)
C9	0.0636 (16)	0.0493 (13)	0.0551 (12)	−0.0051 (12)	0.0120 (11)	−0.0082 (11)
C10	0.0482 (14)	0.0597 (14)	0.0708 (15)	0.0019 (11)	0.0097 (12)	−0.0091 (13)
C11	0.0501 (14)	0.0606 (14)	0.0681 (14)	−0.0077 (12)	0.0211 (11)	−0.0114 (12)
C12	0.0674 (17)	0.0595 (14)	0.0639 (14)	−0.0038 (13)	0.0216 (12)	0.0052 (12)
C13	0.0487 (13)	0.0610 (14)	0.0592 (13)	−0.0004 (11)	0.0113 (11)	−0.0035 (12)
C14	0.0691 (19)	0.103 (2)	0.101 (2)	0.0138 (16)	0.0129 (16)	0.0256 (18)
C15	0.0692 (18)	0.105 (2)	0.116 (2)	−0.0129 (16)	0.0455 (16)	−0.0076 (18)
C16	0.095 (2)	0.0770 (18)	0.0887 (18)	−0.0019 (16)	0.0175 (16)	0.0204 (15)

### Geometric parameters (Å, °)

**Table:**

O1—C5	1.226 (2)	C7—H7B	0.9700
N1—C5	1.329 (2)	C8—C13	1.391 (3)
N1—C4	1.477 (2)	C8—C9	1.395 (3)
N1—H1N	0.8310	C9—C10	1.386 (3)
C1—C4	1.508 (3)	C9—C16	1.517 (3)
C1—H1A	0.9600	C10—C11	1.371 (3)
C1—H1B	0.9600	C10—H10	0.9300
C1—H1C	0.9600	C11—C12	1.370 (3)
C2—C4	1.513 (3)	C11—C15	1.510 (3)
C2—H2A	0.9600	C12—C13	1.383 (3)
C2—H2B	0.9600	C12—H12	0.9300
C2—H2C	0.9600	C13—C14	1.511 (3)
C3—C4	1.512 (3)	C14—H14A	0.9600
C3—H3A	0.9600	C14—H14B	0.9600
C3—H3B	0.9600	C14—H14C	0.9600
C3—H3C	0.9600	C15—H15A	0.9600
C5—C6	1.507 (3)	C15—H15B	0.9600
C6—C7	1.517 (3)	C15—H15C	0.9600
C6—H6A	0.9700	C16—H16A	0.9600
C6—H6B	0.9700	C16—H16B	0.9600
C7—C8	1.503 (3)	C16—H16C	0.9600
C7—H7A	0.9700		
			
C5—N1—C4	126.81 (16)	C8—C7—H7B	109.1
C5—N1—H1N	116.8	C6—C7—H7B	109.1
C4—N1—H1N	116.1	H7A—C7—H7B	107.9
C4—C1—H1A	109.5	C13—C8—C9	118.8 (2)
C4—C1—H1B	109.5	C13—C8—C7	120.6 (2)
H1A—C1—H1B	109.5	C9—C8—C7	120.6 (2)
C4—C1—H1C	109.5	C10—C9—C8	119.6 (2)
H1A—C1—H1C	109.5	C10—C9—C16	118.9 (2)
H1B—C1—H1C	109.5	C8—C9—C16	121.4 (2)
C4—C2—H2A	109.5	C11—C10—C9	122.2 (2)
C4—C2—H2B	109.5	C11—C10—H10	118.9
H2A—C2—H2B	109.5	C9—C10—H10	118.9
C4—C2—H2C	109.5	C12—C11—C10	117.4 (2)
H2A—C2—H2C	109.5	C12—C11—C15	121.7 (2)
H2B—C2—H2C	109.5	C10—C11—C15	121.0 (2)
C4—C3—H3A	109.5	C11—C12—C13	122.8 (2)
C4—C3—H3B	109.5	C11—C12—H12	118.6
H3A—C3—H3B	109.5	C13—C12—H12	118.6
C4—C3—H3C	109.5	C12—C13—C8	119.2 (2)
H3A—C3—H3C	109.5	C12—C13—C14	119.3 (2)
H3B—C3—H3C	109.5	C8—C13—C14	121.5 (2)
N1—C4—C1	110.16 (18)	C13—C14—H14A	109.5
N1—C4—C3	106.71 (16)	C13—C14—H14B	109.5
C1—C4—C3	109.3 (2)	H14A—C14—H14B	109.5
N1—C4—C2	109.41 (18)	C13—C14—H14C	109.5
C1—C4—C2	110.9 (2)	H14A—C14—H14C	109.5
C3—C4—C2	110.2 (2)	H14B—C14—H14C	109.5
O1—C5—N1	123.68 (19)	C11—C15—H15A	109.5
O1—C5—C6	121.82 (19)	C11—C15—H15B	109.5
N1—C5—C6	114.49 (17)	H15A—C15—H15B	109.5
C5—C6—C7	113.87 (17)	C11—C15—H15C	109.5
C5—C6—H6A	108.8	H15A—C15—H15C	109.5
C7—C6—H6A	108.8	H15B—C15—H15C	109.5
C5—C6—H6B	108.8	C9—C16—H16A	109.5
C7—C6—H6B	108.8	C9—C16—H16B	109.5
H6A—C6—H6B	107.7	H16A—C16—H16B	109.5
C8—C7—C6	112.32 (17)	C9—C16—H16C	109.5
C8—C7—H7A	109.1	H16A—C16—H16C	109.5
C6—C7—H7A	109.1	H16B—C16—H16C	109.5
			
C5—N1—C4—C1	−54.9 (3)	C7—C8—C9—C16	−1.2 (3)
C5—N1—C4—C3	−173.5 (2)	C8—C9—C10—C11	−0.9 (3)
C5—N1—C4—C2	67.3 (3)	C16—C9—C10—C11	−179.6 (2)
C4—N1—C5—O1	−1.4 (3)	C9—C10—C11—C12	0.1 (3)
C4—N1—C5—C6	178.5 (2)	C9—C10—C11—C15	178.5 (2)
O1—C5—C6—C7	−6.5 (3)	C10—C11—C12—C13	0.6 (3)
N1—C5—C6—C7	173.65 (19)	C15—C11—C12—C13	−177.8 (2)
C5—C6—C7—C8	179.5 (2)	C11—C12—C13—C8	−0.4 (3)
C6—C7—C8—C13	88.1 (2)	C11—C12—C13—C14	178.8 (2)
C6—C7—C8—C9	−90.9 (2)	C9—C8—C13—C12	−0.5 (3)
C13—C8—C9—C10	1.1 (3)	C7—C8—C13—C12	−179.47 (19)
C7—C8—C9—C10	−179.89 (18)	C9—C8—C13—C14	−179.6 (2)
C13—C8—C9—C16	179.8 (2)	C7—C8—C13—C14	1.4 (3)

### Hydrogen-bond geometry (Å, °)

**Table:**

*D*—H···*A*	*D*—H	H···*A*	*D*···*A*	*D*—H···*A*
N1—H1N···O1^i^	0.83	2.17	2.979 (2)	165

Symmetry codes: (i) *x*, −*y*+1/2, *z*−1/2.

## References

[bb1] Bernstein, J., Davis, R. E., Shimoni, L. & Chang, N.-L. (1995). *Angew. Chem. Int. Ed. Engl.* **34**, 1555–1573.

[bb2] Bruker (2001). *SAINT* . Bruker AXS Inc., Madison, Wisconsin, USA.

[bb3] Bruker (2005). *APEX2* Bruker AXS Inc., Madison, Wisconsin, USA.

[bb4] Sheldrick, G. M. (2003). *SADABS* University of Göttingen, Germany.

[bb5] Sheldrick, G. M. (2008). *Acta Cryst.* A**64**, 112–122. 10.1107/S0108767307043930 18156677

[bb6] Spek, A. L. (2009). *Acta Cryst* D**65**, 148–155. 10.1107/S090744490804362X PMC2631630 19171970

